# Efficacy of *Penicillium limosum* Strain AK-7 Derived Bioactive Metabolites on Antimicrobial, Antioxidant, and Anticancer Activity against Human Ovarian Teratocarcinoma (PA-1) Cell Line

**DOI:** 10.3390/microorganisms11102480

**Published:** 2023-10-03

**Authors:** Dhanyakumara Shivapoojar Basavarajappa, Shaik Kalimulla Niazi, Asmatanzeem Bepari, Rasha Assad Assiri, Syed Arif Hussain, Muzaheed, Sreenivasa Nayaka, Halaswamy Hiremath, Muthuraj Rudrappa, Bidhayak Chakraborty, Anil Hugar

**Affiliations:** 1 P.G. Department of Studies in Botany, Karnatak University, Dharwad 580003, Karnataka, India; dhanyakumara@kud.ac.in (D.S.B.); hmhalaswamy2063@gmail.com (H.H.); rmuthuraj20@gmail.com (M.R.); pallabchakraborty3@gmail.com (B.C.); anilh5157@gmail.com (A.H.); 2 Department of Preparatory Health Sciences, Riyadh Elm University, Riyadh 12611, Saudi Arabia; 3 Department of Basic Medical Sciences, College of Medicine, Princess Nourah bint Abdulrahman University, Riyadh 11671, Saudi Arabia; ambepari@pnu.edu.sa (A.B.); raassiri@pnu.edu.sa (R.A.A.); 4 Respiratory Care Department, College of Applied Sciences, Almaarefa University, Riyadh 13713, Saudi Arabia; syhussain@mcst.edu.sa; 5 Department of Clinical Laboratory Science, College of Applied Medical Sciences, Imam Abdulrahman bin Faisal University, Dammam 31441, Saudi Arabia; marasheed@iau.edu.sa

**Keywords:** *Penicillium limosum*, GC-MS analysis, biological activity, anticancer, apoptosis

## Abstract

Natural metabolites from beneficial fungi were recognized for their potential to inhibit multidrug-resistant human and plant fungal pathogens. The present study describes the isolation, metabolite profiling, antibacterial, and antifungal, antioxidant, and anticancer activities of soil fungi. Among the 17 isolates, the AK-7 isolate was selected based on the primary screening. Further, the identification of isolate AK-7 was performed by 18S rRNA sequencing and identified as *Penicillium limosum* (with 99.90% similarity). Additionally, the ethyl acetate extract of the *Penicillium limosum* strain AK-7 (AK-7 extract) was characterized by Fourier Transform Infrared Spectroscopy (FTIR) and a Gas Chromatography-Mass Spectroscopy (GC-MS) analysis, and the results showed different functional groups and bioactive metabolites. Consequently, a secondary screening of antibacterial activity by the agar well diffusion method showed significant antibacterial activity against Gram-negative and Gram-positive bacterial pathogens. The AK-7 extract exhibited notable antifungal activity by a food poisoning method and showed maximum inhibition of 77.84 ± 1.62%, 56.42 ± 1.27%, and 37.96 ± 1.84% against *Cercospora canescens*, *Fusarium sambucinum* and *Sclerotium rolfsii* phytopathogens. Consequently, the AK-7 extract showed significant antioxidant activity against DPPH and ABTS^•+^ free radicals with IC_50_ values of 59.084 μg/mL and 73.36 μg/mL. Further, the anticancer activity of the AK-7 extract against the human ovarian teratocarcinoma (PA-1) cell line was tested by MTT and Annexin V flow cytometry. The results showed a dose-dependent reduction in cell viability and exhibited apoptosis with an IC_50_ value of 82.04 μg/mL. The study highlights the potential of the *Penicillium limosum* strain AK-7 as a source of active metabolites and natural antibacterial, antifungal, antioxidant, and anticancer agent, and it could be an excellent alternative for pharmaceutical and agricultural sectors.

## 1. Introduction

The emergence of resistance in disease-causing microbes of humans and plants increased the urge to find new and safer alternative treatments. The use of synthetic drugs in pursuing the health of humans and plant diseases has resulted in several side effects, environmental pollution, and a lowering of productivity among different crops [1]. The recent standpoint in the medical and agricultural sector is to use eco-friendly methods, which are noted for fewer side effects and pollution. Natural products formed by different beneficial microbes (bacteria, actinomycetes, fungi, and insects) have received much attention due to their efficiency in synthesizing active secondary metabolites and have accounted for fewer side effects [2]. Among the microbes, fungi were known to produce a class of metabolites with different biological applications. According to an estimation, about 16,000 metabolites from fungal origin contributed 41% of the total 34,000 described metabolites from the other microbes [3].

Natural bioactive fungal metabolites have been recognized for their various applications, such as antimicrobial, antifungal, and anticancer properties. Among the different fungal strains, the genus *Penicillium* (soil, endophyte, and marine-derived) has received much attention because of its potential therapeutical applications. Petit et al. (2009) identified the antimicrobial compounds from soil-derived *Penicillium* sp. *Penicillium chrysogenum* IFL1 showed antibacterial activity against *Staphylococcus aureus*, *Pseudomonas aeruginosa*, and *Bacillus cereus* [4]. Nord et al. (2019) isolated active antibacterial metabolites from *Penicillium spathulatum* Em19 and showed a broad spectrum of antibacterial activity against Gram-negative and Gram-positive bacterial pathogens [5].

The increased virulence of fungal phytopathogens to chemical control methods is a significant issue in agricultural practices. The designing and processing of biological and eco-friendly control of plant pathogens through natural metabolites is of much concern. The metabolites from fungi, especially from the *Penicillium* genus, have been well-documented as excellent alternatives to chemical treatments. Several *Penicillium* sp. were also recognized for their antifungal potential against different plant pathogens. *P. chrysogenum* F-24-28 exhibited antifungal action against *Alternaria alternata*, *Alternaria solani*, *Fusarium culmorum*, *Fusarium sporotrichioides*, and *Fusarium avenaceum* plant pathogens [6]. The metabolites produced from two *Penicillium* spp. showed a broad spectrum of antifungal potential, with 40 to 68% of mycelia inhibition against *Macrophomina phaseolina*, *Cladosporium cladosporioides*, *Fusarium solani*, *Fusarium oxysporum*, and *Apostichopus japonicus var aculeatu* [7]. *P. chrysogenum* IFL1 exhibited antifungal potential against plant pathogens such as *F. oxysporum* [8].

Human diseases such as atherosclerosis, diabetes, asthma, and neurodegenerative disorders are associated with variations in a free radical profile. Synthetic antioxidants were reported as inducers of several side effects in the ailments of the stated diseases. Natural antioxidant metabolites (mycochemicals) are better and safer alternatives to chemically derived metabolites [7]. The natural metabolites from *Penicillium* species with quinines, phenylpropanoids, phenols, flavonoids, and alkaloids have excellent antioxidant properties. Sikandar et al. (2020) reported significant antioxidant activity in *P. chrysogenum* (Snef1216) [9].

Cancer is a multifactor disease that causes severe health issues in humans and has become the second leading cause of morbidity and mortality [10]. Ovarian cancers (gynecologic malignancies) are the seventh leading cause of death globally among the different cancers. The severity of human ovarian teratocarcinoma (PA-1) is characterized by its asymptomatic nature at the initial stages. For most affected patients, it went undetected, and for two-thirds of ovarian-cancer-affected patients, it was detected at the third and fourth stages [11]. According to an estimation, 0.152 million deaths and 0.239 active PA-1 cases have been recorded, and it has also been predicted that 0.371 million incidences and 0.254 million deaths will occur by 2035 [12]. The current strategies for treating ovarian cancer include radiation therapy, chemotherapy, hormonal therapy, debulking surgery, and immunotherapy. However, the medication based on these treatments causes several side effects, reduces the quality of life in affected patients, and high medication costs [13,14]. The challenge of managing ovarian cancer evoked the scientific research field to discover novel and natural therapeutics with fewer side effects.

Many fungi are known to produce a plethora of metabolites and are recognized for their anticancer activities [15]. According to estimation, more than 100 anticancer metabolites are isolated from 50 different fungal strains exhibiting potential activity against 45 human cancer cell lines [16]. Anticancer fungal metabolites can induce cell death by necrosis and apoptosis by instigating morphological changes and reducing cell growth in breast cancer, erythroleukemia, gastric carcinoma, and hepatocellular carcinoma cell lines [17,18]. The authors also reported that *Microsporum* sp. showed effective anticancer properties by inducing DNA fragmentation, the downregulation of *Bcl*-2 expression, the upregulation of *Bax* expression, and reactive oxygen species against human chondrosarcoma, hepatocellular carcinoma, pancreatic duct cancer, and HeLa cell lines, which finally exhibited apoptosis and cell death [19,20].

Likewise, a different genus of fungi, the genus *Penicillium*, has gained consideration because of its anticancer metabolite profile. A study by Tijith et al. (2019) revealed that *Penicillium setosum* has the potential metabolites recognized for anticancer activities [21]. In a recent survey, *Penicillium janthinellum* exhibited anticancer potential, with an IC_50_ value of 44.23 μg/mL against the UMG87 cancer cell line [22]. In another study, *Penicillium citrinum* showed potential antitumor action against human colon carcinoma (HT-29) and non-small lung cancer (A549) cell lines [23]. Jouda et al. (2016) isolated anticancer metabolites penialidin A-C, p-hydroxyphenylglyoxalaldoxime, citromycetin, and brefelfin A from *Penicillium* sp. CAM64, which offered significant cytotoxicity against the HeLa cell line [24]. The strains *Penicillium* sp. NRC F1 and *Penicillium* sp. NRC F16 exhibited anticancer activity against human colon and breast cancer cell lines [25]. In another study, *Penicillium chrysogenum* showed different bioactive secondary metabolites, which exhibited strong antitumor activities against the colorectal carcinoma (Caco-2) cell line [26]. Keeping the noteworthy literature of the *Penicillium* genus, the current study demonstrates the isolation of soil fungi, primary screening, secondary metabolite profiling, and antibacterial, antifungal, antioxidant, and anticancer potential of the soil-derived fungi *Penicillium limosum* strain AK-7.

## 2. Materials and Methods

### 2.1. Collection of Samples, Chemicals, Pathogens, and Cancer Cell Line

The different soil samples were collected from the Dandeli region (latitude 15°19′50″ N and longitude 74°45′53″ E) Uttara Kannada, Karnataka, India. All the required chemicals were purchased from Himedia laboratories, Mumbai, India. Bacterial pathogens were procured from Microbial Type Culture Collection (MTCC), Chandigarh, India, and different phytopathogens were received from the Department of Plant Pathology, University of Agricultural Sciences (UAS), Dharwad, Karnataka, India. The human ovarian teratocarcinoma (PA-1) cell line was procured from the National Centre Cell Science (NCCS) Pune, India. For the anticancer study, fluorescein isothiocyanateannexin-V (Biosciences, Bozeman, MT, USA), propidium iodide (Biosciences), and Doxorubicin (#D1515, Sigma-Aldrich, private limited, Bangalore, Karnataka) Pune, Maharashtra, India were collected.

### 2.2. Isolation of Fungi and Primary Screening

The collected soil samples were transferred to the laboratory using sterilized polyethylene bags, and the isolation of fungi was carried out according to Aziz and Norazwina, (2018) by serial dilution method [27]. The collected soil sample was diluted up to five times (10^−5^) in two replicates. Briefly, 50 g of soil sample was thoroughly mixed with 85% NaCl, and dilutions were prepared by transferring 9 mL of the prepared soil-NaCl mixture from the 1st vial to the 5th vial. Finally, 0.1 mL of dilution from each vial was spread on individual Petri dishes containing Potato dextrose Agar (PDA) medium and sabouraud agar (SDA) medium, and Petri dishes were incubated at 28 °C for 7 days. After the incubation, the subculture and pure culture techniques were performed to obtain the individual fungal colony. The primary screening of antibacterial activity of fungal isolates was performed against selected human bacterial pathogens by cross streak method by streaking the isolates at 90 °C and incubated for 7 days at 30 °C ; after the incubation, the test organisms were inoculated and kept overnight at 37 °C . Finally, on the basis of zone of inhibition (mm), the activity was graded into weak (<25% inhibition), moderate (25–50% inhibition), good (>50% inhibition), and no activity (0% inhibition). The antifungal activity against phytopathogens was performed by dual culture method. The fungal isolates were streaked in the middle of Petri dishes containing PDA media for the dual culture method. A mycelia disc of phytopathogens was placed at both ends, and Petri dishes were placed horizontally to the fungal isolates. Then, the Petri dishes were incubated at 28 °C for 7 days, and the percentage of inhibition was calculated using the following formula [28].

Percentage of inhibition of growth of pathogen = R1 − R2/R1 × 100

(R1 = Growth of fungal phytopathogens in control and R2 = Growth of pathogens in Dual culture plate).

### 2.3. Morphological and Molecular Characterization

Morphological characterization of all the isolates was performed by observing the texture, color of aerial and substrate mycelium, pigment production, and sporulation by using a compound microscope (OLYMPUS CX23, Tokyo, Japan) and the most potent isolate AK-7 was observed for the pattern of spore chain by scanning electron microscopy (JSM-IT500, JEOL, Kyoto, Japan). Molecular characterization of the isolate AK-7 was performed by extracting the DNA from the 12 days PDA-grown culture using a spin column kit (Himedia, Mumbai, India). The purification of 18S rRNA was performed by exonuclease-I shrimp alkaline phosphatase (Exo-SAP), and by using ABI 3500xl, genetic analyzer sequencing was performed. The amplification of sequence through Polymerase Chain Reaction (PCR) was performed in a thermal cycler (Applied Biosystems 2720, thermal Cycler, Foster, CA 94404, USA) using universal primers (1391f and EukBr). The PCR products were validated with 1% agarose gel by using a 1 kb size DNA ladder as a reference. The final 18S rRNA product was subjected to BLAST analysis to find the most similar sequences, and obtained AK-7 sequence were submitted to the NCBI Genbank website with an accession number. Finally, the phylogenetic tree was constructed using the neighbor-joining method using most similar fungal strains and MEGA (7.0) software [24].

### 2.4. Preparation of Ethyl Acetate Extract of Isolate AK-7

Ethyl acetate is the most selected solvent for secondary metabolite extraction due to its high polarity, low boiling point, low toxicity, and selectivity, and it can dissolve hydrophilic and lipophilic compounds [19]. The most potent fungal isolate, AK-7, was selected to extract the secondary metabolites using ethyl acetate. Active culture of isolate AK-7 was grown for 21 days on PDB liquid medium. Then, an equal amount of culture filtrate was mixed with ethyl acetate (1:1) in a separating funnel and kept for 24 h with the intermediate shaking. After 24 h, the metabolite layer (upper) was separated and dried on the watch glass. The collected, dried extract (AK-7 extract) was utilized for further study [28].

### 2.5. Fourier Transform Infrared Spectroscopy (FTIR) Analysis

In the identification of functional groups present in AK-7 extract, the FTIR analysis was performed according to Bhat et al. (2020) [29]. Briefly, the prepared AK-7 extract was subjected to dry at 45 °C for 24 h in a thermostatted desiccator, and the dried extract was mixed with potassium bromide (KBr). Finally, a thin disc of the mixture was prepared, and analysis was performed by FTIR instrument (Nicolet 6700, Thermo Fisher Scientific, Waltham, MA, USA) at the range 400 cm^−1^ and 400 cm^−1^.

### 2.6. Gas Chromatography and Mass Spectroscopy (GC-MS) Analysis

The identification of the chemical composition of AK-7 extract was performed by GC-MS analysis using Shimadzu QP 2010 Ultra instrument. Helium was utilized as carrier gas with a flow rate of 1.2 mL/min, and 70 eV of electron ionization energy was used. The interface temperature was set to 240 °C and 250 °C . A split-less injection with a ratio of 1:10 and injector temperature of 250 °C was used. The 1 µL of AK-7 extract was injected in a column Rxi-5Sil MS (0.25 µm 30 mm ×0.25 µm of thickness). The oven temperature increased from 80 °C to 280 °C with a hold time of 2 min. Data analysis was performed using the GC-MS software, version 3.2 with the 50–800 amu of mass ranges and a scan speed of 0.3 scan/sec. The identification of individual compounds was performed by comparing the mass spectra with the Admas, US National Institute of Standards and Technology (NIST 11, USA), and WILEY 8 mass spectra [30].

### 2.7. Antibacterial Activity by Well Diffusion Method

The antimicrobial activity was performed by well diffusion method against human pathogens such as Gram-negative *Pseudomonas aeruginosa* (MTCC 9027), *Shigella flexneri* (MTCC 1457), *Escherichia coli* (MTCC 40), Gram-positive *Staphylococcus aureus* (MTCC 6908), *Bacillus cereus* (MTCC 11778), and *Bacillus subtilis* (MTCC 6633). The antimicrobial potential was performed through swabbing of mentioned pathogens on freshly prepared Muller–Hinton agar media, and about 6 mm wells were made by using a sterilized cork borer. Then, a different concentration (25, 50, 75, and 100 μL) of AK-7 extract was introduced into separate wells, and plates were incubated for 12 h at 37 °C . After the incubation, the inhibition zones were measured. All the inhibition zones of different concentrations of the AK-7 extract were compared with standard Streptomycin, and for negative treatment, distilled water was used [31].

### 2.8. Antifungal Activity by Food Poisoning Method

The secondary screening of antifungal activity (food poisoning method) of AK-7 extract was performed by adding the AK-7 extract to medium. The AK-7 extract of 100 µL was prepared using sterilized distilled water and added into the autoclaved PDA media after cooling (at 45 °C ) and mixed thoroughly to obtain uniform distribution of the extract. Then, 5 mm of mycelia plugs of 7 days PDA-grown phytopathogens were inoculated individually at the center of Petri dishes. Finally, the Petri dishes were incubated at 28 °C for 7 days, and the inhibition rate (%) was calculated using the following formula [32].

MGI = (DC − DT)/DC × 100

where, MGI: Mycelial growth inhibition rate (%), DC: Diameter of the control samples, DT: Diameter of the test samples.

### 2.9. Antioxidant Activity

The 2, 2-diphenyl-1-picryl-hydroxyl-(DPPH) free radical scavenging assay of AK-7 extract was evaluated according to Basavarajappa et al. (2022) [33]. Briefly, the different concentrations of AK-7 extract (25, 50, 75, 100, and 125 μg/mL) and standards were prepared. Different concentrations of the sample (AK-7 extract) were amended with 2 mL of DPPH (100 μM), and the reaction mixture was made 3 mL by adding methanol. The incubation of the reaction mixture was performed at room temperature for 45 min. After the incubation, the DPPH scavenging activity was analyzed using a spectrophotometer (Shimadzu UV-1800, Tokyo, Japan) by measuring absorbance at 517 nm. The ABTS^•+^ (2, 2′-azino-bis (3-ethylbenzothiazoline-6-sulfonic acid) free radical scavenging assay of AK-7 extract was performed according to the procedure Basavarajappa et al. (2022) [33]. The process began with preparing ABTS^•+^ free radicals (by mixing 7 mM of ABTS^•+^ with 2.45 mM potassium persulphate), and the absorbance of ABTS^•+^ was set to 0.700 ± 0.025 (at 734 nm) to obtain a blue-green appearance. Then 20 μL of the sample (AK-7 extract) from the different concentrations (25, 50, 75, 100, and 125 μg/mL) was added to ABTS (1.0 mL), and finally, the decrease in the absorbance was measured after the 2 min of reaction. The Vit-C (Ascorbic acid) for DPPH and BHA (Butylated hydroxyanisole) for ABTS^•+^ were used as standards. IC_50_ values of AK-7 extract and standards were calculated using the following equation.

% of scavenging activity= 1 − absorbance of sample/absorbance of control × 100

### 2.10. Anticancer Activity (MTT Assay)

The cytotoxicity effect of AK-7 extract was evaluated against the Human ovarian teratocarcinoma (PA-1) cell line. The assay began with the subculturing of the PA-1 cell line on Dulbecco’s Modified Eagle Medium (DMEM, #AL111, Himedia, Mumbai, India). The cell suspension of PA-1 was seeded in a 96-well plate at the cell density of 20.000/well and allowed for 24 h for sufficient growth. Further, different concentrations of AK-7 extract (12.5, 25, 50, 100 and 200 µg/mL) and Doxorubicin at 3 μM/mL as standard was added and the incubation was carried out in high CO_2_ atmosphere (5%) for 24 h at 37 °C . After incubation, the spent media was discarded, and a total volume of 200 µL per well of MTT reagent was added. Then, plates were wrapped with aluminum to avoid light exposure and incubated for 3 h. After the incubation, the MTT reagent was removed, and 100 µL Dimethyl Sulfoxide (DMSO) was added. Then gentle stirring was carried out using a gyratory shaker to dissolve MTT formazan crystals, and finally, the optical density was recorded at 570 nm and 630 nm using an ELISA reader (ELX-800, BioTek, Winooski, VT, USA), and the percentage of cell viability was calculated. The potential of the extract to inhibit the cancer cell growth by 50% is the IC_50_ value. The IC_50_ value of the AK-7 extract against human ovarian teratocarcinoma (PA-1) cell line was determined using linear equation between the log concentration and % of human ovarian teratocarcinoma (PA-1) cell line, and inhibition was calculated using the following equations [31].

% cell viability = OD of treated cells/OD of untreated cells × 100

Y = Mx + C

where, Y: 50, M and C values were obtained from viability graph.

### 2.11. Apoptosis Assay

The AK-7 extract was tested for its effect on the cell death rate in the PA-1 cancer cell line. The PA-1 cell line with a cell density of 0.5 × 10^−6^ cells/2 mL was seeded in a 96-well plate and incubated for 24 h at 37 °C in a CO_2_ incubator. Then, after incubation, the cells were treated with IC_50_ concentration of AK-7 extract and kept for 24 h. The cells with no treatment were considered as control with only 2 mL of media. Then, treated cells were washed twice using PBS (phosphate buffered saline), and PBS was discarded after adding 200 μL of EDTA (trypsin-ethylenediaminetetraacetic acid) and incubated for 3 to 4 min at 37 °C . Further culture medium (2 mL) was added, then treated cells were harvested using polystyrene tubes (12 × 75 mm), and centrifugation was performed at 25 °C at 300 rpm. Finally, the supernatant was removed, and cells were subjected to PBS wash twice after PBS was removed. The 5 µL of Annexin V (Fluorescein isothiocyanate) was added to obtain a pellet with vortex. Incubation was performed at 25 °C for 15 min in dark conditions, followed by the addition of propidium iodide (5 µL) and 1× binding buffer (400 µL), and apoptosis assay was performed using BD FACSCalibur flow cytometer [34,35,36].

### 2.12. Data Analysis

The results of the antibacterial, antifungal, antioxidant, and anticancer experiments were obtained from the three independent trials (n = 3), and graphical representation of each experiment was expressed as mean ± standard deviation.

## 3. Results

### 3.1. Isolation of Soil Fungi

**Figure 1:**
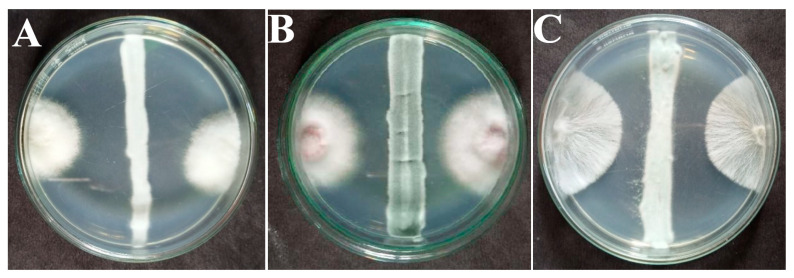
Antifungal activity of isolate AK-7 against phytopathogens: (**A**) *C. canescens*; (**B**) *F. sambucinum*; and (**C**) *S. rolfsii*.

A total of 17 fungal isolates (AK-1 to AK-17) were recovered from the collected soil sample. All the isolates were identified at the genus level by observing the mycelia and sporulation using a compound microscope (OLYMPUS CX23, Tokyo, Japan), and results showed that five isolates, AK-1, AK-2, AK-5, AK-6, and AK-8, belong to the genus *Aspergillus* (29%), three isolates, AK-3, AK-4, and AK-10, belong to *Talaromyces* (17%), four isolates, AK-7, AK-9, AK-13, and AK-14, exhibited characteristic features of *Penicillium* (23%), three isolates, AK-11, AK-12, and AK-17, showed the morphology of *Trichoderma* (17%), and two isolates, AK-15 and AK-16, had *Fusarium* (11%) morphological features. All the isolates were primarily screened for their antimicrobial potential against six selected microbial pathogens. Out of 17 isolates, 8 (47%) isolates (AK-1, AK-2, AK-5, AK-6, AK-9, AK-12, AK-13, and AK-16) exhibited inhibitory effects against at least one tested pathogen, and 5 (29%) isolates (AK-3, AK-4, AK-8, AK-10, and AK-15) were active against four pathogens. Four (23%) isolates (AK-7, AK-11, AK-14, and AK-17) showed inhibition against all tested pathogens (Table 1). Among the isolates, AK-7 was observed to have the highest potential against all pathogens. All 17 isolates were screened for their primary antifungal activity by a dual culture assay against three selected phytopathogens. Among the isolates, 16 (94%) showed inhibition against at least one phytopathogen, isolate AK-7 exhibited a broad spectrum of antifungal potential against all the phytopathogens (Figure 1A–C), and isolate AK-7 was selected for further screening based on its antimicrobial and antifungal potential.

### 3.2. Morphological and Molecular Characterization

**Figure 2:**
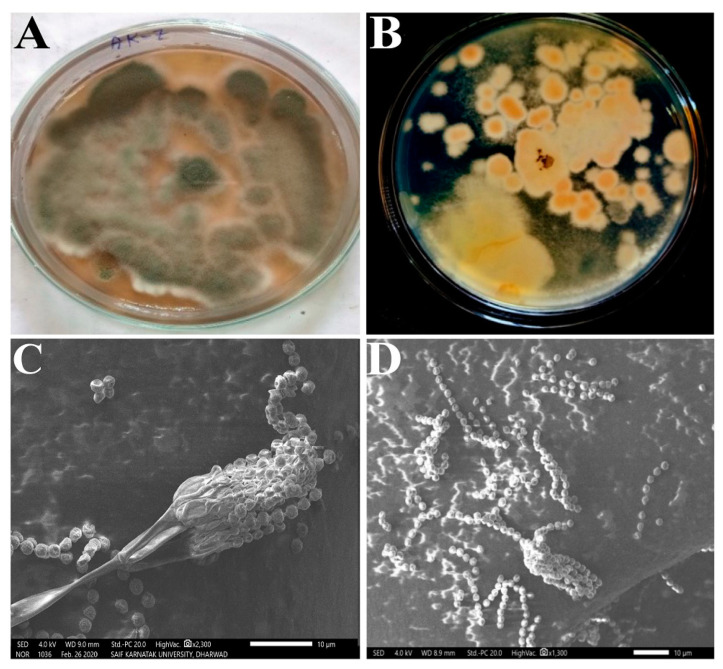
Morphological characterization of isolate AK-7: (**A**) Aerial mycelia; (**B**) Substrate mycelia; (**C**) Microscopic visualization of mycelia and sporulation; and (**D**) SEM image showing conidiophores and conidia.

**Figure 3:**
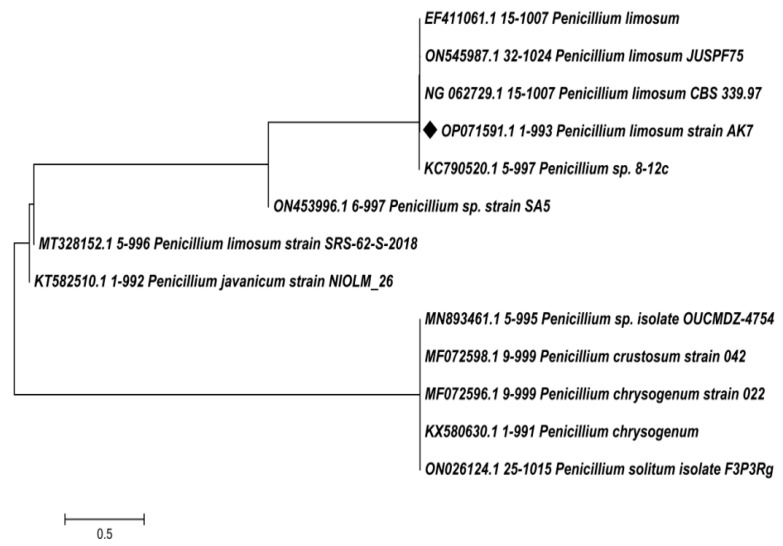
Dendrogram showing phylogenetic relationship of *Penicillium limosum* strain AK-7.

Morphologically, isolate AK-7 exhibited an aerial greenish mat-like appearance and brownish-orange substrate mycelium. A highly branched septate mycelium that bears dense sporulation with spherical conidiophores at the end of the mycelium branch. The spherical-shaped conidiophores showed phialide, which bears a brush-like appearance. The SEM analysis of the spore chain exhibited biverticilate conidiophores, divergent mutulae, ampulliform phialides, and globose conidia (Figure 2A–D). These characteristics confirmed that isolate AK-7 belongs to *the Penicillium* genus. Molecular characterization of the isolate AK-7 was performed through the 18S rRNA sequencing, and a total of 993 base pairs were subjected to BLAST analysis with the accession number (OP071591). The isolate AK-7 showed the highest similarity, 99.90%, with *Penicillium limosum* CBS 339 (Accession number-NG 062729). Further, the phylogenetic tree was constructed with the most similar sequences, and isolate AK-7 exhibited an evolutionary relationship with *Penicillium limosum* CBS 339 (Accession number-NG 062729) by forming a lateral branch (Figure 3). Finally, isolate AK-7 was identified as *Penicillium limosum* strain AK-7.

### 3.3. FTIR Analysis

**Figure 4:**
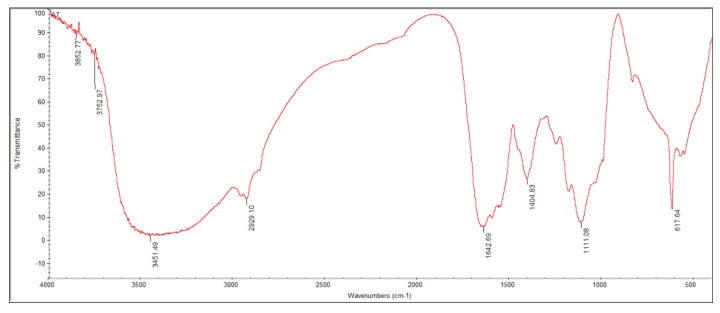
FTIR analysis of AK-7 extract showing different functional groups.

The detection of functional groups present in ethyl acetate extract of isolate *Penicillium limosum* strain AK-7 (AK-7 extract) was carried out by FTIR analysis. Results revealed peaks at 3852, 3752, 3451, 2929, 1642, 1404, 1111, and 617 cm^−1^ (Figure 4). The weak peaks at 3852 and 3752 and the broad peak at 3451 depicted the OH stretching alcohol, and the sharp peaks at 2929 and 1642 revealed the presence of C-H stretching alkanes and C=C stretching alkene. The strong peaks obtained at 1404 and 1111 indicated S=O stretching sulfate and C=O stretching secondary alcohol. The sharp peak at 617 depicted the C=Br stretching halo compounds.

### 3.4. GC-MS Analysis

**Figure 5:**
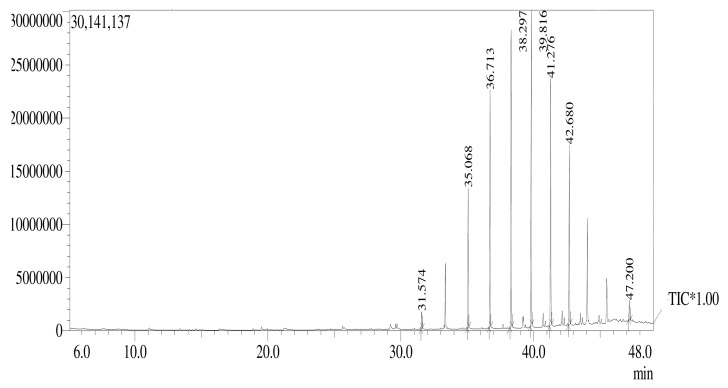
GC-MS chromatogram of AK-7 extract.

The chemical compounds present in the AK-7 extract were identified by GC-MS analysis, and results revealed eight major peaks with different retention times (Figure 5). The major compounds in the chromatogram with the highest percentage of the area showed Celidoniol, Deoxy- (21.03%), Heneicosane (20.34%), Etratetracontane (18.30%), Eicosane (15.67%), Hexatriacontane (12.51%), Tricosane (9.28%), Pentatriacontane (1.71%), and Nonadecane (1.16%). The molecular formula and molecular weight of the obtained compounds are displayed in Table 2. The GC-MS spectrum of the AK-7 extract was compared with the National Institute of Standards and Technology (NIST).

### 3.5. Antibacterial Activity by Well Diffusion Method

**Figure 6:**
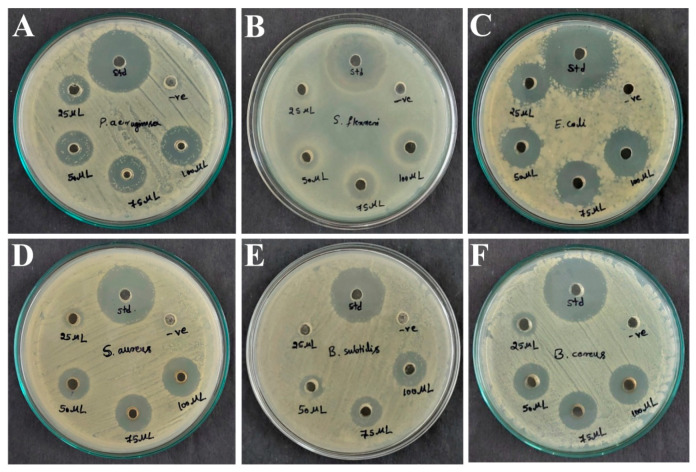
Antimicrobial activity of AK-7 extract by agar well diffusion method: (**A**) *P. aeruginosa*; (**B**) *S. flexneri*; (**C**) *E. coli*; (**D**) *S. aureus*; (**E**) *B. subtilis*; and (**F**) *B. cereus*.

**Figure 7:**
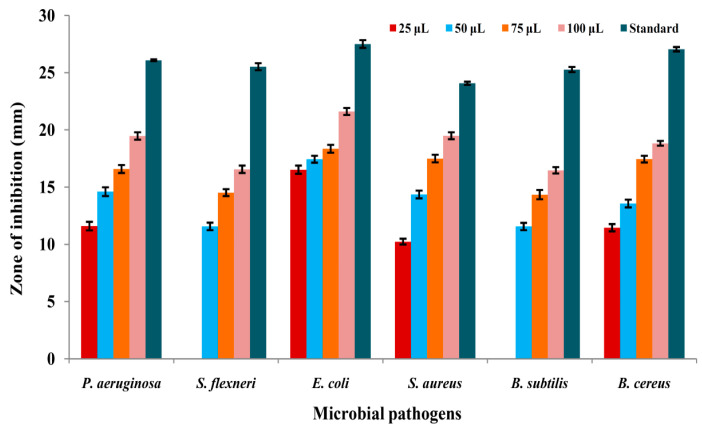
Graphical representation of antibacterial activity of AK-7 extract. (The inhibition zones represent the mean of triplicates trials and expressed as ± standard deviation).

The antibacterial activity of AK-7 extract was tested via the agar well diffusion method, and results showed concentration-dependent inhibition against tested human pathogens (Figure 6A–F). The maximum activity was found against *E. coli* with 21.60 ± 0.30 mm of inhibition at 100 μL followed by 19.48 ± 0.30 mm, 19.46 ± 0.36 mm, and 18.82 ± 0.20 mm of inhibition against *S. aureus*, *P. aureginosa*, and *B. cereus* at 100 μL of concentration. A moderate antibacterial potential was exhibited against 16.55 ± 0.32 mm and 16.46 ± 0.28 mm of inhibition against *S. flexneri* and *B. subtilis* at the concentration of 100 μL. No activity was observed against *S. flexneri* and *B. subtilis* at 25 μL of concentration. A graphical representation of antibacterial activity is depicted in Figure 7.

### 3.6. Antifungal Activity by Food Poison Method

**Figure 8:**
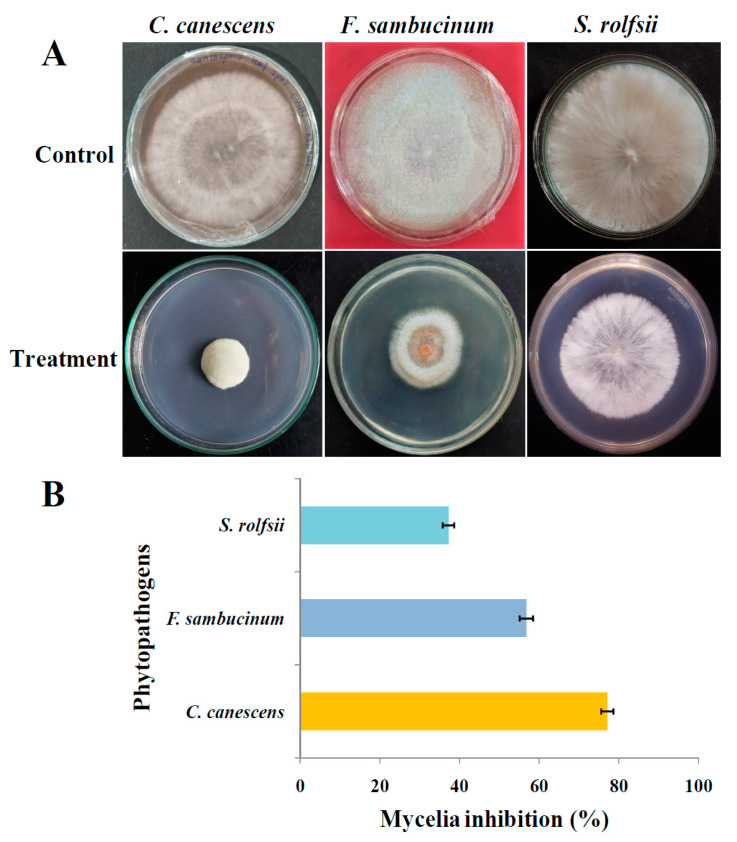
Antifungal activity of AK-7 extract by food poison method: (**A**) Control and treated agar plates with AK-7 extract; and (**B**) Graphical representation of antifungal activity of AK-7 extract. (The percentage of inhibition rate represent mean of the triplicate trials and expressed as ± standard deviation).

The food poisoning method was performed by evaluating the antifungal potential of AK-7 extract against three phytopathogens (Figure 8A). The results showed antifungal potential with a maximum inhibition rate of 56.84 ± 1.62% against *F. sambucinum* at a 100 μL concentration. A considerable inhibition rate was observed with 48.42 ± 1.27% and 55.96 ± 1.84% against *C. canescens* and *S. rolfsii* at the 100 μL concentrations in comparison with the growth of mycelia in control plates are graphically represented in Figure 8B.

### 3.7. Antioxidant Activity

**Figure 9:**
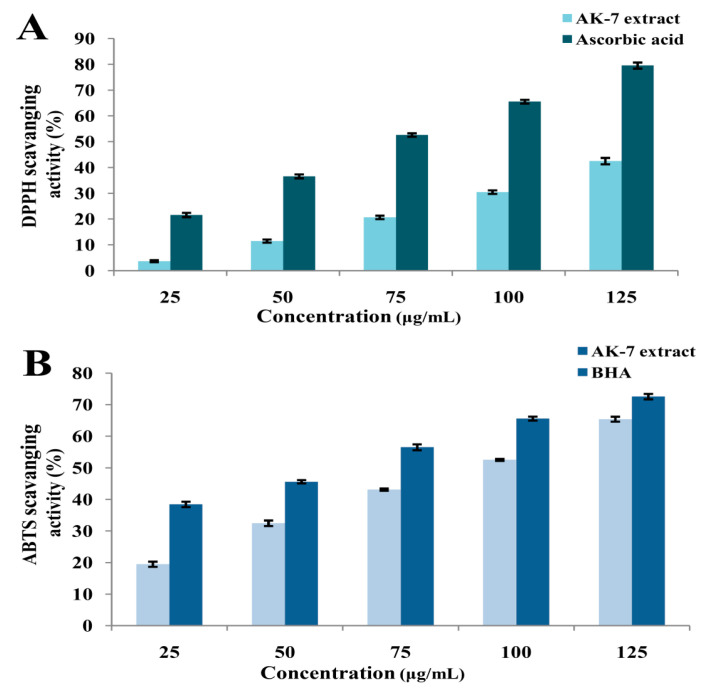
Antioxidant activities of AK-7 extract: (**A**) DPPH free radical scavenging assay and (**B**) ABTS^•+^ free radical scavenging assay. (The percentage of scavenging activity represent mean of the triplicate trials and expressed as ± standard deviation).

The AK-7 extract showed a progressive increase in antioxidant activity, with an increase in concentration against DPPH and ABTS^•+^ free radicals. A significant reduction in the DPPH free radical exhibited 3.69 ± 028%, 11.47 ± 0.3%, 20.65 ± 0.32%, 30.45 ± 0.31%, and 42.50 ± 0.36% at the concentrations of 25, 50, 75, 100, and 125 μg/mL of the AK-7 extract. The standard ascorbic acid showed 21.83 ± 0.83%, 36.25 ± 0.76%, 52.25 ± 0.67%, 62.20 ± 0.75%, and 78.53 ± 1.17% scavenging activity at the concentrations of 25, 50, 75, 100, and 125 μg/mL (Figure 9A). The IC_50_ values obtained were 59.084 μg/mL for the AK-7 extract and 14.844 μg/mL for the ascorbic acid. The AK-7 extract showed potent antioxidant potential against ABTS^•+^ free radicals, with 19.47 ± 0.81%, 32.79 ± 0.87%, 42.77 ± 0.35%, 52.56 ± 0.33%, and 65.10 ± 0.78% at the concentrations of 25, 50, 75, 100, and 125 μg/mL. The standard BHA exhibited a 38.11 ± 0.86%, 45.18 ± 0.54%, 56.20 ± 0.92%, 65.28 ± 0.63%, and 72.60 ± 0.85% reduction in ABTS^•+^ free radicals at the concentrations of 10, 20, 30, 40, and 50 μg/mL (Figure 9B). The AK-7 extract showed an IC_50_ of 73.362 μg/mL, and BHA exhibited 23.269 μg/mL.

### 3.8. Anticancer Activity (MTT Assay)

**Figure 10:**
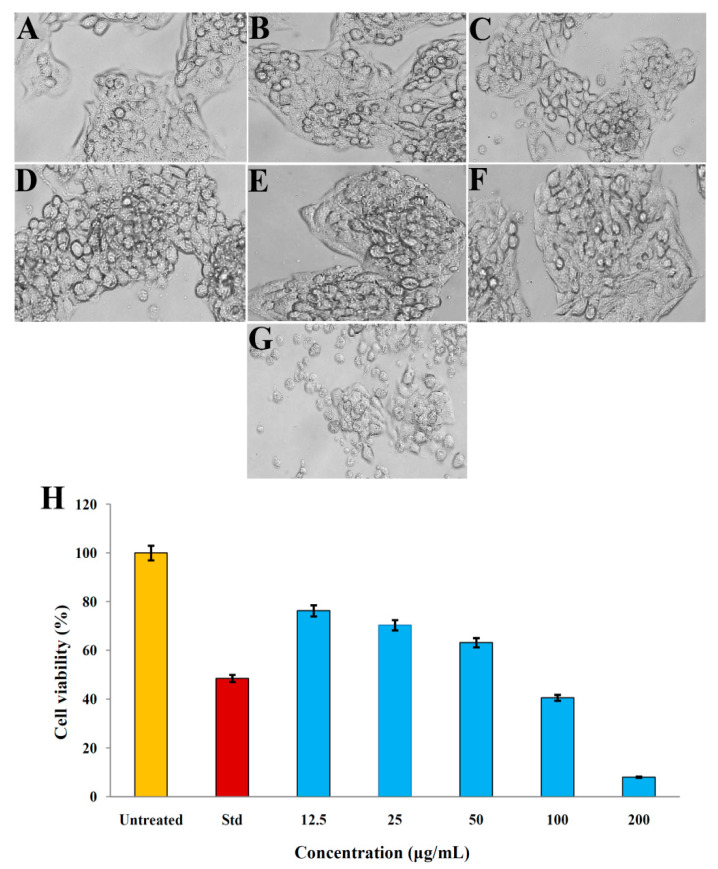
Anticancer activity (MTT assay) of AK-7 extract against PA-1 cell line: (**A**) Untreated control; (**B**) Standard Doxorubicin; (**C**) 12.5 μg/mL; (**D**) 25 μg/mL; (**E**) 50 μg/mL; (**F**) 100 μg/mL; (**G**) 200 μg/mL; and (**H**) Comparative graphical representation on cell viability of different concentration of AK-7 extract. (The cell viability percentage of each treatment represents mean of the triplicates trials and expressed as ± standard deviation).

The AK-7 extract was subjected to evaluate its anticancer potential against the human ovarian teratocarcinoma (PA-1) cell line through an MTT assay. Results showed a significant decrease in the cell viability of the PA-1 cell line with the increased concentrations of the AK-7 extract. Cell viability of 76.24 ± 0.31%, 70.32 ± 0.25%, 63.17 ± 0.36%, 40.53 ± 0.42%, and 8.01 ± 0.19% was observed at the concentrations of 12.5, 25, 50, 100, and 200 μg/mL of the AK-7 extract, respectively. Doxorubicin, as a standard control, exhibited 48.48 ± 0.01% cell viability, and further AK-7 extract had an IC_50_ of 82.04 μg/mL against the PA-1 cell line (Figure 10A–H).

### 3.9. Apoptosis Assay

**Figure 11:**
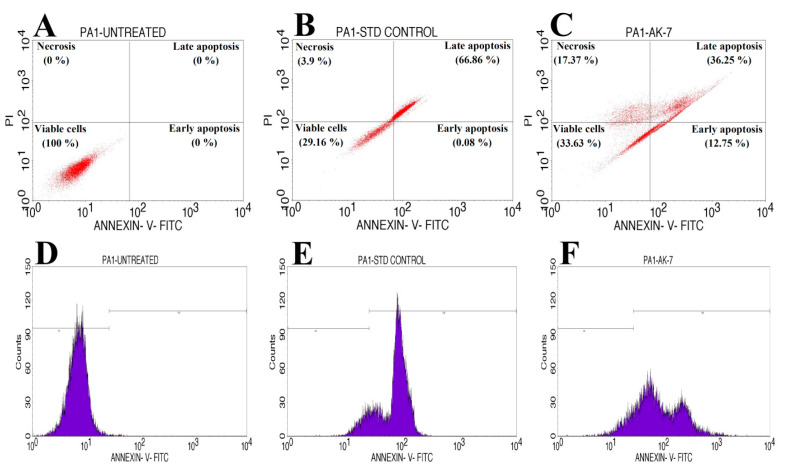
Flow cytometric analysis of PA-1 cell line treated with AK-7 extract. The quadrangular plots indicate Annexin V/PI expression on PA-1 cell line: (**A**) Untreated cell line; (**B**) Standard treated cell line; and (**C**) AK-7 extract treated cell line. Cell cycle analysis of PA-1 cell line; (**D**) Untreated cell line; (**E**) Standard treated cell line; and (**F**) AK-7 extract treated cell line.

The apoptosis assay of the AK-7 extract was performed through the Annexin V/PI expression method against the PA-1 cell line using an IC_50_ concentration of 82.04 μg/mL. The viable and early apoptosis cells were observed by positive Annexin V/PI staining, and the negative staining of cells with PI indicated late apoptosis and necrosis (Figure 11A–F). The PA-1 cells treated with the IC_50_ concentration of the AK-7 extract showed early apoptosis (12.75%), late apoptosis (36.25%), necrosis (17.37%), and viable cells (33.63%). The standard Doxorubicin exhibited early apoptosis (0.08%), late apoptosis (66.86%), necrosis (3.9%), and viable cells (29.16%), and untreated PA-1 cell lines showed 100% viable cells. Further, cell cycle progression (M1 phase) and cell cycle arrest (M2 phase) in the AK-7-extract-treated PA-1 cell line was observed, with a 65.42% cell cycle arrest and 10.46% cell cycle progression, where standard Doxorubin-treated cells showed 71.13% and 9.67% cell progression and cell arrest, respectively. The untreated PA-1 cell line exhibited a cell progression of 0.28% and cell arrest of 78.70%.

## 4. Discussion

Soil-derived fungi were much-recognized organisms due to their secondary metabolites profile. Many fungal species are excellent producers of antibiotics, antimicrobial, antifungal, and anticancer metabolites [27]. Among the several soil fungi, the *Penicillium* species are ubiquitous due to their distribution in a wide range of habitats and attracted most of the researcher’s interest for the synthesis, evaluation, and screening of valuable and active secondary metabolites in various pharmaceutical and medical fields. The present report explores the isolation of soil fungi, secondary metabolite profiling, and antimicrobial, antifungal, antioxidant, and anticancer potential. Among the 17 fungal isolates, isolate *Penicillium limosum* strain AK-7 (AK-7) was selected for its maximum activity against human microbial pathogens and phytopathogens in the primary screening. Further, the AK-7 was selected for its secondary metabolite profiling and secondary screening of antibacterial, antifungal, and anticancer activity against the human ovarian teratocarcinoma (PA-1) cell line.

Morphological observations through a compound microscope and SEM analysis showed that the *Penicillium limosum* strain AK-7 exhibited a radial sulcate colony floccose surface with a light greenish appearance. The results were strongly supported by the earlier reports; the *P. limosum* (CNUFC DMS3-17) isolated from soil morphologically showed aerial greenish to grey appearance and orange-brownish substrate mycelia with white mycelia, sulcate texture, floccose, and moderately dense sporulation with biverticilate conidiophores. Molecular characterization through 18S rRNA and phylogenetic analysis suggested evolutionary relationships with most similar types of sequences. The *Penicillium limosum* strain AK-7 shared the phylogeny with *P. limosum* CBS 339. The morphological observation is the prominent fundamental and basic technique to identify the fungal species at the genus level, and 18S rRNA sequencing was the most preferred molecular technique, which exhibits a high resolution of molecular identification of the fungal isolates up to the species level [35]. A similar study was conducted using the 18S rRNA gene sequence technique to identify *P. citrinum* and *Penicillium* CAM64 [23,24].

The FTIR analysis of the AK-7 extract clearly hinted at the presence of various functional groups, such as primary and secondary alcohols, alkanes, alkenes, sulfate, and halo compounds. The results can be compared with the FTIR analysis of *P. italicum*, which showed the presence of different functional groups such as amide, alkenes, alkyl halides, alkane, and sulfate [37]. FTIR analysis is the non-destructive and accurate method used for the rapid identification of a wide range of functional groups, and basic chemical profiles of the bioactive metabolites present in microorganisms [38]. GC-MS analysis of AK-7 extract exhibited a total of eight compounds with significant peaks, which include five alkanes (Nonadecane, Tricosane, Eicosane, Heneicosane, and Celidoniol, Deoxy-) and three hydrocarbons (Tetratetracontane, Hexatriacontane, and Pentatriaconate). Earlier studies reported that Nonadecane and Heneicosane compounds from the isolated fungi were recorded for antibacterial and cytotoxicity effects [39]. In another report, the authors identified bioactive metabolites such as Eicosane, Tetratetracontane, and Tricosane from three *Penicillium* Spp. reported for antibacterial, antifungal, and anticancer potential [40]. The secondary metabolites play a vital role in constituting various biological activities (antioxidant, antibacterial, antifungal, and anticancer). Grijseels et al. (2017) reported a broad spectrum of bioactive secondary metabolite production from the *P. nalgiovense* and *P. vulpinum* [41].

The AK-7 extract exhibited significant antibacterial potential against *E. coli* (21.60 ± 0.30 mm) followed by *S. aureus* (19.48 ± 0.30 mm) and *P. aureginosa* (19.46 ± 0.36 mm) and dose-dependent activity to rest of the pathogens. AK-7 extract exhibited significant variation in the inhibitory potential against both Gram-positive and Gram-negative pathogens. The antibacterial activity can be assumed by the action of secondary metabolites, which cause lysis of the plasma membrane and distort its function in pathogens, leading to the pathogen’s eventual death. The effect of metabolites on the permeability of the pathogen cell membrane by releasing active ions (K^+^ and PO^4-^) can induce membrane leakage and affect the functional level of the bacterial enzymes [29]. The membrane of Gram-negative bacteria comprises lipopolysaccharide molecules and phospholipids; these molecules can tolerate antimicrobial drugs by various modes (limiting drug uptake, inactivation of drug, and modification in targeted drug). Interestingly, the AK-7 extract exhibited a notable inhibitory effect on Gram-negative bacteria that hinted at the bioactive metabolites of the extract might be involved in suppressing the pathogen’s growth with possible mechanisms. The results were in accordance with an earlier report where *Penicillium* sp. exhibited strong antibacterial activity against human pathogens [42]. In another study, Nurulita et al. (2019) reported a broad spectrum of antibacterial activity of *Penicillium* sp. LBKURCC34 extract against Gram-positive and Gram-negative human pathogens [43]. The antibacterial activity is directly correlated with the metabolites. In the present study, The AK-7 extract revealed the potential metabolites Nonadecane, Celidoniol Deoxy, Tetratetracontane, and Tricosane, which are known for their significant antibacterial potential. Hence, these metabolites may be involved in the inhibition of tested pathogens [41,44,45].

The antifungal potential of AK-7 was evaluated against three selected phytopathogens. Strong antifungal activity was noted against *F. sambucinum*, followed by *S. rolfsii* and *C. canescens* in both dual culture and food poison assay. Antifungal results can be compared with the report of Karpova et al. (2021); in their study, isolated *Penicillium chrysogenum* F-24-28 exhibited significant antifungal activity against nine tested phytopathogens [6]. The antifungal potential of *Penicillium expansum* against different phytopathogens suggested potent mycelia inhibition by producing antifungal metabolites [46]. It was observed that the effect of the extract on the mycelia of the fungal pathogens causes a series of detrimental effects, such as alterations in mycelia morphology through disruption, loss of membrane integrity, which leads to cellular material leakage and, finally, cell death. Antifungal potential can be induced by the production of hydrolytic enzymes such as amylase, protease, chitinase, glucanases, and degradation of the fungal cell wall, lysis, and hyphal collapse, which affect mycelia germination of plant pathogens [7]. In the previous reports, compounds such as Eicosane, Tetratetracontane, Tricosane, Hexatriaconthane, and Pentatriaconate demonstrated antifungal activity against diverse fungal pathogens. As a result, in the present report, the AK-7 extract hints at the presence of these potential metabolites that can be attributed to the antifungal activities [40,47]. In the present study, the AK-7 organism and extract exhibited significant mycelial inhibition through mycelia destruction of tested phytopathogens.

Significant antioxidant potential of the AK-7 extract against DPPH and ABTS^•+^ free radicals was observed and hinted at the increased reduction in the respective radicals and increased concentration. The results have consisted of the DPPH scavenging potential (81.3%) of *Penicillium expansum* isolated from soil [48]. In another study, *Penicillium brefeldianum* F4a exhibited potent antioxidant activity against ABTS^•+^ free radicals [49]. The contents of phenolics, flavonoids, polyphenols, vitamin C, and carotenoids play a vital role in scavenging free radicals by donating hydrogen atoms or electron transfer [50]. In the present context, the antioxidant metabolites from AK-7 extract might be involved in reducing the DPPH and ABTS^•+^ free radicals. The antioxidant potential of the microbial extract is related to the presence of secondary metabolites with scavenging potential against free radicals. Hence, in the present report, the antioxidant activity of the AK-7 extract displayed metabolites such as Tetratetraconthane, Heneicosane, Tricosane, Eicosane, and Pentatriaconthane, which are known for the antioxidant nature of different free radicals (DPPH, ABTS, etc.) [41,51].

The AK-7 extract exhibited dose-dependent anticancer activity against the human ovarian teratocarcinoma (PA-1) cell line. The gradually increased anticancer potential was observed with the increased extract concentration from 12.5 to 200 μg/mL, and a significant reduction in cell viability was noted from 76.24 ± 0.31% at 12.5 μg/mL and reached the lowest 8.01 ± 0.19% at the concentration of 200 μg/mL with the IC_50_ value of 82.04 μg/mL. A similar anticancer study was performed by Waill et al. (2022); they reported the significant anticancer potential of the *Penicillium* sp. NRC F1 and *Penicillium* sp. NRC F16 against human colon and human breast cancer cell lines [25]. In a recent study, *Penicillium citrinum* Thom. exhibited anticancer potential against the A549 and MCF-7 cell lines with the IC_50_ values of 280.7 and 283.0 μg/mL and showed a significant reduction in cell viabilities [52]. Anticancer activity is the ability of the compounds present in the microbial extract to exhibit antitumor potential. The AK-7 extract hinted at the presence of antitumor compounds such as Heneicosane, Nonadecane, Celidoniol Deoxy, hexatriaconthane, Pentatriaconthane, and Tricosane and these compounds are documented for the antitumor activities in the earlier reports. Hence, the anticancer activity of AK-7 extract against the human ovarian teratocarcinoma (PA-1) cell line may be attributed to the anticancer action of these metabolites [53,54,55].

The apoptosis induction (eventual cell death) in the AK-7 extract-treated PA-1 cell line was studied by Annexin V apoptosis detection procedure. Results showed effective induction of early apoptosis, late apoptosis, and necrosis induction in IC_50_ (82.04 μg/mL) concentration treated PA-1 cell line. A significant shifting of cells to early, late apoptosis and maximum necrosis of the PA-1 cells (33.63%) suggested that AK-7 extract could induce apoptosis. The results were similar to the earlier reports where *Penicillium sclerotiorum* extract treated A549, A431, U251, and HeLa cancer lines showed a significant reduction in cell viability and increased eventual cell death (apoptosis) at the IC_50_ concentrations of 10, 20, 32 and 7.75 μg/mL [56]. Apoptosis can be instigated by the effective action of the drug, which causes DNA fragmentation, prevention of DNA replication, breaking of DNA double-strand (through inactivation of topoisomerase II), ROS generation (reactive oxygen species), affect the mitochondrial functioning level, and finally, damage of DNA which leads to the apoptosis [57,58,59,60]. In the present context, AK-7 extract significantly induced and influenced the apoptosis mechanism in the PA-1 cell line through a series of mechanisms that finally led to mitochondrial dysfunction and cell death compared to the untreated cell line. Venkatachalam et al. (2019) observed increased apoptosis in HepG2, HeLa, and MCF-7 cancer cell lines treated with *Penicillium rubens* extract; they also observed upregulation of *Bcl-2*, *Bax*, and p53 expressions levels significantly increased the apoptotic efficacy [61]. It was evident from the literature that induced cancer cell death is the result of potential metabolites present in the extracts. In correlation with the present study, compounds such as Nonadecane, Tricosane, Celidonial Deoxy, and Pentatriaconthane were observed for apoptosis-inducing capabilities, which led to a series of events in cancer cells that resulted in cell death [62]. Interestingly, the literature also revealed the significant role of these metabolites recorded from the fungal extract in the induction of apoptosis in different cancer cell lines, such as MCF-7, COLO 205, HeLa, HEK 293, and A-431 [63].

## 5. Conclusions

The current study demonstrated the characterization and effectiveness of ethyl acetate extract of soil-derived fungi *Penicillium limosum* strain AK-7 in various biological activities. The characterization AK-7 ethyl acetate extract hinted at the different functional groups, including a rich profile of alkanes, alkenes, and alcoholic functional groups in FTIR analysis. GC-MS analysis hinted at the multifunctional secondary metabolites Nonadecane, Eicosane, Celidonial, Deoxy, Tricosane, and Pentatriaconthane with antimicrobial, antifungal, antioxidant, and antitumor activities. Further antibacterial activity of AK-7 extract showed that it has the ability to inhibit both Gram-positive and Gram-negative bacteria, which suggested the importance of its capability in clinical research. The antifungal potential in both dual culture and food poison methods suggested that the AK-7 extract has the potential to inhibit the growth of mycelia in three tested phytopathogens and hinted at the agricultural application as an antifungal agent. Consequently, the AK-7 extract evidenced the dose-dependent scavenging potential (DPPH and ABTS^•+^ free radicals) with the IC_50_ concentrations of 59.084 μg/mL and 73.362 μg/mL, which suggested the natural antioxidant potential. Cytotoxicity of AK-7 extract against the human ovarian teratocarcinoma (PA-1) cell line displayed dose-dependent activity and, at the IC_50_ concentration of 82.04 μg/mL, witnessed the capability to instigate apoptosis (eventual cell death) at lower concentrations. Finally, it was concluded that the ethyl acetate extract of *Penicillium limosum* AK-7 has multiple potentials and might be used as a future natural and safer commercial drug in the agricultural field and in pharmaceutical therapies.

## Figures and Tables

**Table 1:** Primary screening of antibacterial activity of fungal isolates.

Fungal Isolates	Pathogens					
	*P. aeruginosa*	*S. flexneri*	*E. coli*	*S. aureus*	*B. subtilis*	*B. cereus*
AK-1	-	-	-	-	+	-
AK-2	-	-	-	+	-	-
AK-3	-	+	-	+	+	+
AK-4	+	-	-	+	+	+
AK-5	-	-	-	+	-	-
AK-6	-	-	-	-	-	+
AK-7	+++	+++	+++	+++	+++	+++
AK-8	-	-	+	+	+	+
AK-9	-	-	-	-	+	-
AK-10	+	+	-	-	+	+
AK-11	++	+	++	++	+++	++
AK-12	-	-	-	-	+	-
AK-13	-	-	+	+	+	+
AK-14	+	++	+++	++	++	++
AK-15	-	+	+	-	+	+
AK-16	-	-	-	-	+	-
AK-17	++	+++	++	++	+++	++

Note: + = Weak activity, ++ = Moderate activity, +++ = Good activity, − = No activity.

**Table 2:** Compounds obtained from GC-MS analysis of AK-7 extract.

Peak No.	Retention Time	Area %	Compound Name	Molecular Formula	Molecular Weight
1	37.514	1.16	Nonadecane	C_19_H_40_	268.5
2	35.068	9.28	Tricosane	C_23_H_48_	324.6
3	36.713	15.67	Eicosane	C_20_H_42_	282.5
4	38.297	20.34	Heneicosane	C_21_H_44_	296.6
5	39.816	21.03	Celidoniol, Deoxy-	C_29_H_60_	408.79
6	41.276	18.30	Tetratetracontane	C_44_H_90_	619.2
7	42.680	12.51	Hexatriacontane	C_36_H_74_	507.0
8	47.200	1.71	Pentatriacontane	C_35_H_72_	492.9

## Data Availability

The data presented in this research are available on request.
